# Correction to: Genome-wide investigation of persistence with methotrexate treatment in early rheumatoid arthritis

**DOI:** 10.1093/rheumatology/kead540

**Published:** 2023-10-12

This is a correction to: Anton Öberg Sysojev, Saedis Saevarsdottir, Lina-Marcela Diaz-Gallo, Gilad N Silberberg, Lars Alfredsson, Lars Klareskog, Eva Baecklund, Lena Björkman, Alf Kastbom, Solbritt Rantapää-Dahlqvist, Carl Turesson, Ingileif Jonsdottir, Kari Stefansson, Thomas Frisell, Leonid Padyukov, Johan Askling, Helga Westerlind, Genome-wide investigation of persistence with methotrexate treatment in early rheumatoid arthritis, *Rheumatology*, 2023, https://doi.org/10.1093/rheumatology/kead301

The originally published version of this manuscript required correction.

Tables 1, 2, S6 and S7 contained information erroneously labelled as the starting dose of MTX. The variable in question, however, is not the starting dose of MTX as was described within the table, but instead describes the ‘package Daily Defined Dosage’, a clinically irrelevant variable.

The paper has now been corrected to remove this information from the affected tables.

